# Cytokine/Chemokine assessment as a complementary diagnostic tool for inflammatory skin diseases

**DOI:** 10.3389/fimmu.2022.1028435

**Published:** 2022-11-16

**Authors:** Timothy J. Liu, Lynlee L. Lin, Erin McMeniman, Jason Wu, Yung-Ching Kao, Snehlata Kumari, Glen M. Boyle, James W. Wells, H. Peter Soyer, Jazmina L. Gonzalez-Cruz

**Affiliations:** ^1^ Dermatology Research Centre, The University of Queensland Diamantina Institute, The University of Queensland, Brisbane, QLD, Australia; ^2^ Department of Dermatology, Princess Alexandra Hospital, Metro South Health, Brisbane, QLD, Australia; ^3^ Department of Cell and Molecular Biology, QIMR Berghofer Medical Research Institute, Brisbane, QLD, Australia

**Keywords:** inflammatory skin diseases, cytokines, chemokines, immunology, diagnostic, skin biopsy

## Abstract

Inflammatory skin conditions are the 4^th^ leading cause of non-fatal health burden in the general population worldwide. The diagnosis of skin lesions due to systemic drug reactions, viral or bacterial exanthems, or in patients with psoriasis, atopic dermatitis or contact dermatitis is often difficult and relies heavily upon conventional histopathologic examination. Conversely, it is widely accepted that the cutaneous profile of inflammatory markers, or ‘inflammatory signature’, is differentially expressed in various skin conditions. In this pilot study, we investigated the possibility of inflammatory skin disease diagnosis from an immunological perspective in small punch biopsies. We collected lesional and perilesional punch biopsies from 139 patients suffering from a variety of inflammatory skin conditions and attending the Dermatology Department at the Princess Alexandra Hospital in Brisbane, Australia. Using bead-based immunoassays we were able to measure 13 out of 17 inflammatory markers from a pre-selected multi-analyte panel and to detect significant differences between lesional and perilesional biopsies from each individual patient. Hierarchical and unbiased clustering methods based on inflammatory signatures grouped psoriasis and atopic dermatitis lesions into individual clusters in contrast to other skin conditions, highlighting the potential of inflammatory signatures to be used as diagnostic differentiators and to inform alternative targets in anti-inflammatory treatment strategies.

## Introduction

Inflammatory skin conditions are highly prevalent. Over 4% of the Australian population suffer from non-malignant skin conditions, amongst which psoriasis, atopic dermatitis, drug eruptions and follicular disorders are the most common (1). As such, these conditions have a wide-reaching impact upon the healthcare system, in outpatient and inpatient settings alike. Within general practice, 17.4 per 100 encounters are for dermatological complaints, of which a significant portion are accounted for by inflammatory dermatoses, including cutaneous infection (22%), dermatitis (12%), unspecified rash (5%) and acne (4%) (2, 3). Similarly, of the 2 - 4.2% of Emergency Department presentations that are for dermatological complaints, common conditions include psoriasis (25%), atopic dermatitis (23%) and cellulitis (15%) (4, 5). Furthermore, 81% of inpatient dermatology consultations alone are made for dermatitis, autoimmunity, drug reaction or infection (6).

Inflammatory skin conditions are a broad grouping of disorders with diverse aetiology. Their triggers include infections, drug-induced hypersensitivity, autoimmunity, physical injury and environmental changes. These conditions involve the pathological activation of innate and adaptive immunity by inflammatory mediators such as cytokines and chemokines, manifesting in local injury to the skin. These immunopathological changes are reflected clinically in terms of rashes (7). The differentiation of any given rash primarily relies on a combination of clinical history and examination, complemented by cutaneous biopsy for confirmative or eliminative purposes (8). Histopathology is the most frequently used diagnostic test in dermatology, forming part of the full evaluation of skin through a collaborative effort between physician and pathologist. Other tests, such as direct immunofluorescence, tissue culture, flow cytometry and patch tests, are collectively of ancillary utility (9).

Often, the clinical diagnosis of inflammatory dermatoses is challenging despite the availability of conventional histopathology and supplementary tests (10, 11). Although some conditions are idiosyncratic, many conditions of contrasting aetiology, such as viral exanthems and drug eruptions, are indistinguishable due to overlapping clinical and histopathological features (12). Drug eruptions, in particular, may imitate virtually all histopathological patterns of inflammation in the skin, including leukocytic infiltrates, cytotoxic epidermal changes, lichenoid dermatitis, spongiosis, granulomatosis, vasculitis, psoriasisform epidermal pattern, vesiculobullous patterns, panniculitis, folliculitis and scleroderma (13). No current diagnostic test can reliably differentiate a drug-induced cause from another process, whether infectious or allergic. Furthermore, both clinical and histopathological examinations, being dependent upon qualitative observations, may be limited by factors such as degree of dermatological and dermatopathological experience as well as correct biopsy technique and collection of a representative lesion (14). Overall, there is a clear need within inflammatory skin diagnosis to develop more reliable and user-independent diagnostic aids.

The present study aimed to explore the potential of an immunological approach to diagnose inflammatory skin lesions. Previous studies examining immunological parameters in the skin have shown an association between different disease types and inflammatory mediator proteins, including cytokines and chemokines (15–21). Whilst certain inflammatory markers are known to associate with specific disease types, it is not known whether disease-specific ‘inflammatory signatures’ can be defined and utilised for the diagnosis of patients. A side-by-side comparison of multiple biomarkers of various conditions is essential to characterise their signatures. In this study, we sought to investigate the feasibility of this multi-analyte approach by using a small punch biopsy to compare inflammatory signatures from a broad array of inflammatory dermatoses. Specifically, our aims were firstly to demonstrate that a multi-analyte panel of inflammatory markers can be measured in a small punch biopsy of skin, and secondly, to establish the correlation of inflammatory signatures according to disease type. To achieve our aims, we selected a set of 17 cytokines/chemokines reported in the literature to be expressed by common inflammatory conditions (**Table 1**). According to our selection, we expected to detect high levels of interleukin (IL)-5, perforin, granzyme B, soluble FAS ligand (sFASL), C-C motif Chemokine Ligand (CCL)-17, transforming growth factor (TGF)-β and IL-4 in drug eruptions; high IL-4, IL-13, IL-21, IL-22, C-X-C motif chemokine ligand (CXCL)-10 and low arginase levels in atopic dermatitis; high levels of TGF-β, C-reactive protein (CRP), CCL5, neutrophil gelatinase-associated lipocalin (NGAL) and adiponectin in contact dermatitis; high CXCL-10 and low IL-22 levels in hidradenitis suppurativa, and high levels of IL-22, IL-13, arginase and vascular endothelial growth factor (VEGF) in psoriasis (15–19, 21–33).

**Table 1:** Disease type discriminatory strategy. Combination of chemokine/chemokine inflammatory signatures expected to be detected in each diagnostic category.

Skin disease type	Cytokine/Chemokine signature	References
Drug eruption	IL-5^high^, Perforin ^high^, Granzyme B ^high^, sFASL ^high^, CCL-17 ^high^, TGF-β ^high^, IL-4^high^	(19, 22–24)
Atopic Dermatitis	IL-4^high^, IL-13 ^high^, IL-21 ^high^, IL-22 ^high^, CXCL-10 ^high,^ Arginase^low^	(17, 20, 21, 25, 26)
Contact dermatitis	TGF-β, CRP, CCL-5, NGAL, Adiponectin	(15, 18, 26, 27)
Hidradenitis suppurativa	IL-22 ^low^, CXCL-10^high^	(28, 29)
Psoriasis	IL-22 ^high^, IL-13^high,^ Arginase^high^, VEGF^high^, CCL-5^high^	(30, 31)
Cutaneous T-cell lymphoma	IL-4^high^, IL-13^high^	(32, 33)

## Methods

A total of 139 patients were recruited in this study from the Department of Dermatology, Princess Alexandra Hospital, Brisbane, Australia. From each patient a pair of 2 mm punch biopsies were collected, including inflamed skin, which we termed ‘lesional’, and nearby normally appearing ‘perilesional’ skin. Demographics, past medical history, medications, clinical photographs, and pathology reports were also recorded. The biospecimens were harvested on ice in protease inhibitor solution, homogenised using metal beads, and then stored at -80°C until use.

BioLegend LEGENDplex bead-based immunoassays were employed to quantitate human inflammatory proteins within the samples. This approach allowed the simultaneous detection of up to 13 analytes in one clinical sample. Two customised BioLegend kits (13-plex and 4-plex) were selected based upon cytokines reported in the literature to be differentially expressed in common inflammatory skin conditions.

Immunoassay results were analysed using the CytExpert Software V2.2 (BioLegend). T-Distributed Stochastic Neighbour Embedding (t-SNE) and PhenoGraph clusteting were performed on R statistical package, using the cytokine/chemokine bead-based array data after data normalization.

## Results

**Figure 1:**
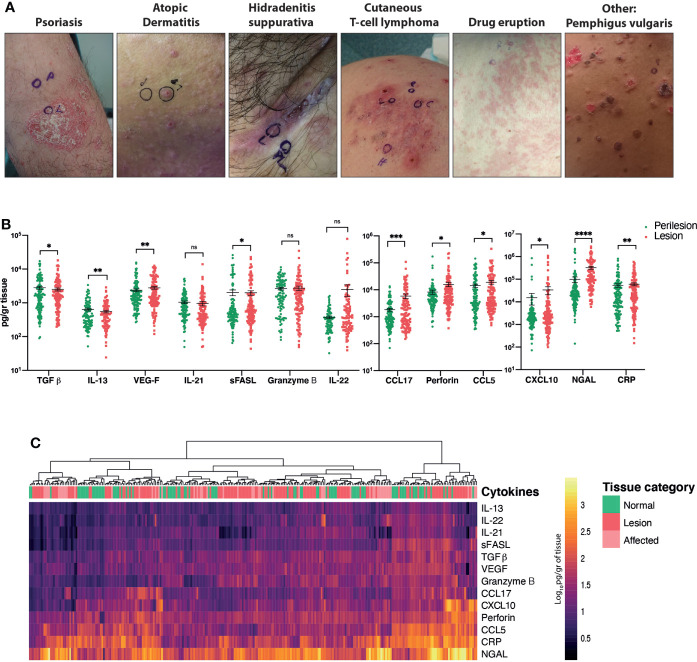
Comparison of the cytokine/chemokine profiles of perilesional and lesional skin obtained from patients with inflammatory skin conditions. **(A)** Representative pictures of the main diagnostic categories. **(B)** Quantification of selected analytes. Wilcoxon matched-pairs signed rank test, *p* < 0.05 (*), *p* < 0.01 (**), *p* < 0.001 (***), *p* < 0.0001 (****). **(C)** Heat map corresponding to log_10_-transformed concentrations. Unsupervised hierarchical clustering performed across samples (columns) and ordered according to similarity of the expression profile by samples. ns, non significant.

The average patient age was 52.3 years and 58% of the participants were male (*n* = 80). At the time of biopsy, ~81% of patients were undergoing treatment for their skin condition (*n* = 114). These treatments included targeted biologics, immunosuppressants, corticosteroids, antihistamines, antibiotics, retinoids, and physical therapies such as phototherapy. Each paired sample was assigned to a diagnostic category (psoriasis (PsO, *n* = 30), atopic dermatitis (AD, *n* = 19), hidradenitis suppurativa (HS, *n* = 12), cutaneous T-cell lymphoma (CTCL, *n* = 11) and drug eruption (*n* = 10)) (**Figure 1A**). Disease types with less than 10 samples (i.e., prurigo nodularis, viral exanthem, tinea corporis, etc) were categorized as ‘Other’ (*n* = 57).

Of the 17 total analytes, we detected 13 cytokines/chemokines, with IL-4 and IL-5 being beneath the detection limit and adiponectin and arginase being above the detection limit (**Figure 1B**).

Wilcoxon matched-pairs signed rank tests revealed significant differences in cytokine/chemokine expression between pooled lesional and perilesional samples (**Figure 1B**). These include TGF-β, CXCL-10, perforin and IL-22 (*p* < 0.05); CRP, VEGF and IL-21 (*p* < 0.01), CCL-17 (*p* < 0.001) and NGAL (*p* < 0.0001) proteins. Notably, a retrospective analysis of the photographs revealed that subtle clinical inflammation (‘affected’) was observable in 36% of the collected perilesional samples, and this was accordingly evident in our measurement of cytokines/chemokines. This fact added to the large variance of cytokine/chemokine expression levels detected across patients and could be responsible for the lack of clear segregation between lesional and perilesional categories when all samples were pooled together (**Figure 1C** **)**.

**Figure 2:**
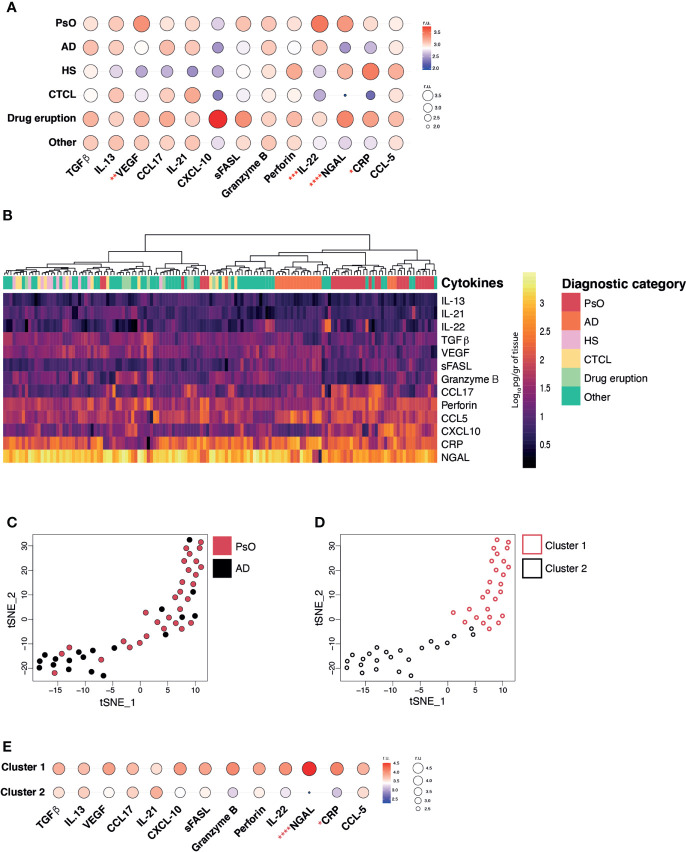
Inflammatory skin disease diagnosis by measuring cytokine/chemokine profiles. **(A)** Balloon plot representing the geometric mean expression values of each analyte across all disease categories (relative units, r.u.). **(B)** Heat map corresponding to the log_10_-transformed concentrations. Unsupervised hierarchical clustering performed across samples (columns) and ordered according to similarity of the expression profile by samples. **(C)** T-Distributed Stochastic Neighbour Embedding (t-SNE) dimensionality reduction plot and unbiased PhenoGraph clustering **(D)** of PsO and AD samples. E. Balloon plot representing the geometric mean expression values of each analyte relative to PhenoGraph clusters (relative units, r.u.). Kruskal-Wallis test **(A)** and Mann-Whitney test **(E)**, *p* < 0.05 (*), *p* < 0.01 (**), *p* < 0.001 (***), *p* < 0.0001 (****).

To evaluate the differentiating markers within our sample set, we examined the mean expression of each cytokine/chemokine per diagnostic category (**Figure 2A**). Even though most lesions were collected from patients undergoing treatment (**Table 2**), we were able to confirm the expression of several disease-specific inflammatory markers in our samples. For instance, AD lesions presented high levels of IL-13, IL-21 and IL-22, HS presented low IL-22, and CTCL presented high IL-13 levels. As expected, drug eruption lesions showed the widest expression range across all analytes, reflecting their heterogenous morphology and immunopathology. In the case of PsO lesions, although characteristic cytokines such as Tumour Necrosis Factor (TNF)-α, IL-17 and IL-23 were not included in our pilot panel, we were able to observe elevations in other relevant cytokines such as NGAL, VEGF and IL-22. The comparison of the mean expression of each cytokine/chemokine across all diagnostic categories, revealed that VEGF, IL-22, NGAL and CRP were differentially expressed, indicating their potential to be used to diagnostically distinguish the present subset of diseases.

**Table 2:** Pharmacological treatment per diagnostic category.

Skin disease type	Treatment
**Psoriasis** (*n* = 30)	- Topical corticosteroid (60.0%) - IL-17A inhibitor (ixekizumab/secukinumab) (13.3%) - Methotrexate (10.0%) - Antibiotic (6.7%) - Antihistamine (6.7%) - IL-23 inhibitor (guselkumab) (6.7%) - Systemic corticosteroid (6.7%) - Phototherapy (3.3%) - Retinoid (3.3%) - Calcineurin inhibitor (3.3%) - IL-12/IL-23 inhibitor (ustekinumab) (3.3%) - JAK inhibitor (tofacitinib) (3.3%) - PD-L1 inhibitor (durvalumab) (3.3%) - TNF-α inhibitor (adalimumab) (3.3%) **Untreated** (13.3%)
**Atopic Dermatitis** (*n* = 19)	- Topical corticosteroid (63.2%) - Antihistamine (36.8%) - Antibiotic (26.3%) - Calcineurin inhibitor (10.5%) - Methotrexate (10.5%) - Systemic corticosteroid (10.5%) - Azathioprine (5.3%) **Untreated** (10.5%)
**Hidradenitis suppurativa** (*n* = 12)	- Antibiotic (58.3%) - Resorcinol (41.7%) - TNF-α inhibitor (adalimumab) (25.0%) - Topical corticosteroid (16.7%) - Systemic corticosteroid (8.3%) **Untreated** (25.0%)
**Cutaneous T-cell lymphoma** (*n* = 11)	- Topical corticosteroid (72.7%) - Calcineurin inhibitor (27.3%) - Phototherapy (27.3%) - Retinoid (27.3%) - Methotrexate (18.2%) **Untreated** (9.1%)
**Drug Eruption** (*n* = 10)	- Antihistamine (50.0%) - Topical corticosteroid (40.0%) - Antibiotic (20.0%) - Systemic corticosteroid (20.0%) **Untreated** (20.0%)

Summary of ratios and treatment types given to patients, stratified according to diagnostic categories.

Subsequently, we questioned whether the cytokine/chemokine patterns generated for each individual lesion had the power to cluster and segregate samples back to their diagnostic categories. To do so, an unsupervised hierarchical clustering, according to similarity of inflammatory signature, was performed by using Euclidean distances across lesional specimens. Based on all 13 analytes PsO and AD lesions tended to cluster together (**Figure 2B** columns) whereas other disease types, such as HS, CTCL, drug eruption and those classified as ‘other’ did not appear to congregate possibly due to high expression variability and insufficient sample numbers. As a proof of concept, we used the t-SNE dimensionality reduction algorithm to evaluate the relationship between the lesional samples within the two largest categories, AD and PsO (**Figure 2C**). Two distinctive clusters containing PsO (Cluster 1), and AD (Cluster 2) were observed and confirmed by the PhenoGraph unbiased clustering method (**Figure 2D**). The major drivers of differentiation between both clusters were the higher expression of NGAL and CRP in Cluster 1 (>PsO) in comparison to Cluster 2 (>AD) (**Figure 2E**). Overall, our results highlight the feasibility of using our selected 13-marker panel in 2 mm skin punch biopsies to differentiate PsO from AD lesions.

## Discussion

Our results showed that an inflammatory signature, drawn from a curated panel of multiple inflammatory markers, can be successfully detected in 2 mm punch biopsies with the BioLegend LEGENDplex bead-based assay. Previous literature had already established the association of upregulated cytokines (as well as the downregulation of others) within certain inflammatory skin diseases, suggesting that rashes may be differentiable by their cytokine levels. The present research establishes a methodology to derive multidimensional quantitative information from small samples, thereby extending the depth of comparisons between disease types. Most notably, by offering quantitative measurement, bead-based protein detection provides information free from interpretive bias and covering a large panel of cytokines and chemokines. By comparison, histopathology primarily involves subjective interpretation, and immunohistochemistry is limited to a semiquantitative assessment of typically one analyte at a time (34). Furthermore, the assessment of a patient’s immunophenotype can occur on 2 mm small punch biopsies by eliminating the need for larger (3 or 4 mm), or repeated biopsy collections.

In this study, we observed correlations between inflammatory signature and disease type, such as PsO and AD, in spite of varying degrees of chronicity and severity. This observation is particularly remarkable given that more than 80% of patients were undergoing treatment, whether it be in the form of topical corticosteroids or systemic immunosuppressants or targeted biologics. On the other hand, it is evident that lesions from other categories, such as HS, CTCL, and drug eruption, did not clearly associate into clusters. There could be several contributing factors to explain this finding. Firstly, the chosen analyte panel may simply not have covered the cytokines/chemokines necessary to delineate these disease types, particularly with relatively small sample sizes. To test this, we will need to increase statistical power through increased sample size and introduce additional key differential analytes to the cytokine/chemokine panel. Secondly, it is likely that in the context of various anti-inflammatory treatments being administered to the majority of patients, correlations will have been weakened. It is worthwhile to acknowledge, however, that patient recruitment in a real-world clinical setting, wherein most patients attending clinic have already commenced some form of treatment, would be severely limited if controlling for treatment status. Therefore, in the scope of the present exploratory study, our endeavour to establish inflammatory signature correlations was best enabled by the recruitment of patients regardless of treatment status. Thirdly, the findings may reflect the inherent heterogenous morphology and immunopathology of certain conditions and their subtypes. For instance, as previously established, drug eruptions are known to morphologically resemble a wide range of skin conditions, both clinically and histopathologically. Likewise, CTCLs are known to exhibit a wide spectrum of clinical, immunological and genetic phenotypes (32), thereby accounting for the observed variation in cytokine/chemokine profiles. Ultimately, although not all disease types could be distinguished using the chosen analyte panel, these results suggest the potential to refine inflammatory signatures pertaining to disease type, eventually leading to diagnostic differentiation by inflammatory markers. The versatility of our approach allows the easy addition or substitution of analytes within the chosen panel, as well as the replacement of bead-based immunoassays with other technologies, such as RNA sequencing, as alternative means to measure cytokine/chemokine expression.

It is notable that across the entire sample set, there was no significant segregation between lesional and perilesional inflammatory signatures. This may be explained by the possibility that in several cases, perilesional skin, despite appearing normal and healthy, contained imperceptible ‘subclinical’ inflammation. This hypothesis is especially feasible in systemic auto-inflammatory diseases such as psoriasis and atopic dermatitis, whereby pro-inflammatory cytokines are not only found to be present within diseased skin, but also elevated in non-affected skin and additionally elevated in the serum (35, 36). One of the strengths of our findings is that the generation of disease cytokine/chemokine signatures can be performed independently from perilesional samples as it is the comparison between lesional signatures that aids their classification. Whilst not pertaining to the aims of the present study, future research comparing inflammatory profiles of healthy skin to non-lesional skin in those with inflammatory skin diseases may elucidate the parameters that drive the maintenance of healthy skin, as opposed to those which produce a pro-inflammatory microenvironment.

The characterisation of disease-specific cytokine and chemokine panels may advance our knowledge of the pathogenesis of inflammatory skin diseases and their clinical subtypes, particularly in rare and poorly understood diseases. It may also be possible to define the immunological progression of chronic diseases such as AD and PsO by analysing their inflammatory signatures in correlation to chronicity, fluctuations in severity and response to treatment. Therefore, we envisage that inflammatory dermatoses will come to be understood not only within the classic paradigm of precise clinical and histopathological descriptions, but furthermore according to characteristic immunological phenotypes. The therapeutic consequence of defining inflammatory signatures has the potential to inform the development of targeted treatments and also to enable those treatments to be tailored according to one’s individual immune response, in line with the concept of personalised medicine.

The demonstration of inflammatory signature detection within samples as small as 2 mm punch biopsies, which themselves are smaller than the standard 3- or 4-mm punch biopsies typically collected for histopathology, suggests a potential route toward minimally invasive biopsy methods. To further minimize biopsy burden and improve logistical efficiency, the time-consuming procedure involved in the collection of punch biopsies may ultimately be replaced by minimally invasive biospecimen collection methods, such as microbiopsies (37) or tape striping (38), without the requirement of local anaesthesia or suturing.

Overall, our pilot study highlights the potential of inflammatory signatures to be used to differentiate inflammatory skin conditions and lead to the creation of new technologies to aid in the diagnostic process. Further characterisation of inflammatory signatures will not only clarify our understanding of inflammatory skin conditions, but further the development and application of anti-inflammatory therapies.

## Data availability statement

The original contributions presented in the study are included in the article material. Further inquiries can be directed to the corresponding author.

## Ethics statement

This study was approved by the Metro South Human Research Ethics Committee (approval #/HREC/18/QPAH/245, 4 June 2018) and The University of Queensland (approval #2018001616, 8 August 2018) and conducted in accordance with the Declaration of Helsinki. Participants provided written consent after receiving a Participant Information and Consent Form. Written informed consent was obtained from the individual(s) for the publication of any potentially identifiable images or data included in this article.

## Author contributions

Conceptualization: LLL, GMB, JWW, HPS, JLGC; Data Curation:, TJL, JLGC; Formal Analysis: TJL, JLGC; Funding Acquisition: JWW, HPS, LLL, JLGC; Investigation: TJL, EKM, JKW, YCK, HPS, JLGC; Methodology: TJL, JWW, JLGC; Project Administration: LLL, JWW, HPS, JLGC; Resources: TJL, EKM, YCK; Software: JLGC; Supervision: LLL, JWW, HPS, JLGC; Validation: TJL, JWW, HPS, SK, JLGC; Visualization: TJL, JLGC; Writing - Original Draft Preparation: TJL, JLGC; Writing - Review and Editing: TJL, SK, JWW, HPS, JLGC.

## Funding

This work was supported an Australian Skin and Skin Cancer Research Centre Enabling Grant (JWW and JLGC) and a Translational Research Institute Spore Grant (HPS, JWW, LLL and JLGC).

## Acknowledgments

This research was carried out at the Translational Research Institute, Woolloongabba, QLD 4102, Australia. We thank the patients and staff at the Princess Alexandra Hospital, Brisbane, Australia, for their participation in this study. HPS holds an NHMRC MRFF Next Generation Clinical Researchers Program Practitioner Fellowship (APP1137127).

## Conflict of interest

The authors declare that the research was conducted in the absence of any commercial or financial relationships that could be construed as a potential conflict of interest.

## Publisher’s note

All claims expressed in this article are solely those of the authors and do not necessarily represent those of their affiliated organizations, or those of the publisher, the editors and the reviewers. Any product that may be evaluated in this article, or claim that may be made by its manufacturer, is not guaranteed or endorsed by the publisher.
